# Conserved cryptic recombination signals in Vκ gene segments are cleaved in small pre-B cells

**DOI:** 10.1186/1471-2172-10-37

**Published:** 2009-06-25

**Authors:** Anne E Lieberman, Masayuki Kuraoka, Marco Davila, Garnett Kelsoe, Lindsay G Cowell

**Affiliations:** 1 Department of Biostatistics and Bioinformatics, Duke University, Durham, NC, USA; 2 Department of Immunology, Duke University, Durham, NC, USA; 3 Department of Medicine, Division of Medical Oncology, Memorial Sloan-Kettering, New York, NY, USA

## Abstract

**Background:**

The cleavage of recombination signals (RS) at the boundaries of immunoglobulin V, D, and J gene segments initiates the somatic generation of the antigen receptor genes expressed by B lymphocytes. RS contain a conserved heptamer and nonamer motif separated by non-conserved spacers of 12 or 23 nucleotides. Under physiologic conditions, V(D)J recombination follows the "12/23 rule" to assemble functional antigen-receptor genes, *i.e*., cleavage and recombination occur only between RS with dissimilar spacer types. Functional, cryptic RS (cRS) have been identified in V_H _gene segments; these V_H _cRS were hypothesized to facilitate self-tolerance by mediating V_H _→ V_H_DJ_H _replacements. At the *Igκ *locus, however, secondary, *de novo *rearrangements can delete autoreactive VκJκ joins. Thus, under the hypothesis that V-embedded cRS are conserved to facilitate self-tolerance by mediating V-replacement rearrangements, there would be little selection for Vκ cRS. Recent studies have demonstrated that V_H _cRS cleavage is only modestly more efficient than V(D)J recombination in violation of the 12/23 rule and first occurs in pro-B cells unable to interact with exogenous antigens. These results are inconsistent with a model of cRS cleavage during autoreactivity-induced V_H _gene replacement.

**Results:**

To test the hypothesis that cRS are absent from Vκ gene segments, a corollary of the hypothesis that the need for tolerizing V_H _replacements is responsible for the selection pressure to maintain V_H _cRS, we searched for cRS in mouse Vκ gene segments using a statistical model of RS. Scans of 135 mouse Vκ gene segments revealed highly conserved cRS that were shown to be cleaved in the 103/BCL2 cell line and mouse bone marrow B cells. Analogous to results for V_H _cRS, we find that Vκ cRS are conserved at multiple locations in Vκ gene segments and are cleaved in pre-B cells.

**Conclusion:**

Our results, together with those for V_H _cRS, support a model of cRS cleavage in which cleavage is independent of BCR-specificity. Our results are inconsistent with the hypothesis that cRS are conserved solely to support receptor editing. The extent to which these sequences are conserved, and their pattern of conservation, suggest that they may serve an as yet unidentified purpose.

## Background

The ability to mount specific immune responses depends on a highly diverse repertoire of T- and B-cell antigen-receptor molecules. The genetic diversity required for millions of distinct antigen-receptors is created by the somatic recombination and fusion of individual variable (V), diversity (D), and joining (J) gene segments in a process known as V(D)J recombination. During V(D)J recombination, genomic DNA is cleaved at the boundaries of individual V, D, and J gene segments and the intervening DNA removed or inverted; subsequently, the newly apposed gene segments are ligated to form the variable region exon of one of the four types of antigen-receptor genes (reviewed in [1]). These recombination events are mediated by RAG-1 and RAG-2 in the form of a V(D)J recombinase holoenzyme that is directed to proper sites of cleavage by DNA motifs known as recombination signals (RS). RS are located at the boundaries of V, D, and J gene segments and defined by highly conserved heptamer- and less-well conserved nonamer sequences that are separated by non-conserved spacer regions 12- or 23-base pairs (bp) in length [2-5]. Under physiologic conditions, V(D)J recombination follows the "12/23 rule" to assemble functional antigen-receptor genes, *i.e*., cleavage and recombination occur only between RS with dissimilar spacer types.

RS-like sequences that are unassociated with V, D, or J gene segments have been identified in the genomes of mice and humans [4,6-18]. A subset of these cryptic RS (cRS) are located within the *Igh *and *Igκ *loci [7-17]. cRS in the *Igh *locus are embedded at the 3' end of V_H _gene segments where they mediate V_H _→ V_H_DJ_H _replacement reactions [7-10,13,15,19]. cRS in the *Igκ *locus are located within introns where they mediate inactivation of *Igκ *alleles [11,17,20-22]. With the exception of [8], previous studies of V-embedded cRS have focused on the *Igh *locus. V_H _gene replacement mediated by V-embedded cRS can rescue the development of B cells bearing autoreactive receptors and has been described as a mechanism for the maintenance of self-tolerance [7,9,22-24]. In fact, it has been argued that V_H _cRS are conserved specifically to provide a mechanism for secondary rearrangements at the *IgH *locus, as "secondary V_H _to J_H _[recombination] cannot work because V_H _and J_H _[RS] do not meet the [12/23] requirement for recombination and because D segments, the guardians of this rule, are deleted by the primary V(D)J recombination" [7].

Previously, we conducted a global analysis of cRS across mouse V_H _gene segments using a computational algorithm to predict the location and functional activity of V_H _cRS; these predictions were then tested using a ligation-mediated PCR (LM-PCR) to detect V_H _cRS cleavage in purified populations of mouse B-lineage cells recovered from murine bone marrow [4,25]. We discovered that not only are cRS conserved at sites distributed throughout V_H _gene segments but also that V_H _cRS are cleaved only during the pro-B cell stage of development [25]. Both results are inconsistent with the paradigmatic view that functional V_H _cRS are maintained to facilitate the rescue of autoreactive B cells that would otherwise be lost to the mechanisms of self tolerance [7,9,22-24]. Our results suggested to us that V_H _cRS may be conserved for other reasons [25].

In contrast to receptor editing via V_H _replacement, receptor editing at the *Igκ *locus, takes the form of either secondary, *de novo *Vκ → Jκ rearrangements that replace or invert primary VκJκ joins [26-30], or more rarely, inactivating rearrangement with cRS that flank the Cκ exon [21]. Secondary, de novo rearrangements are not only possible at the *Igκ *locus, but highly efficient because of the locus' organization: Vκ gene segments are associated with 12-RS while Jκ gene segments are associated with 23-RS, removing the need for a D gene segment and allowing repeated, direct VκJκ rearrangements; Vκ genes are present in both orientations, resulting in many inversion rearrangements and conserving Vκ gene segments that lie between the rearranging Vκ and Jκ gene segments for subsequent rearrangements; The possibility for rearrangement at the *Igλ *locus further increases the opportunity for editing.

A corollary of the argument that V_H _cRS are conserved to provide a mechanism for secondary rearrangement at the *Igh *locus [7,9] is that cRS would not be conserved within Vκ gene segments. Thus far, however, there have been no systematic attempts to search for cRS within Vκ gene segments, to determine the extent of Vκ cRS conservation, or to determine whether they are functional. Previous work searched Vκ sequence alignments for partial heptamer motifs (CACA) at a location within Vκ orthologous to the location of the 3' V_H _cRS [8,9]. It was noted that 10% of the Vκ gene segments examined contain this partial heptamer motif [8]. We extend this study using a computational algorithm that allows for systematic scanning of the full length of Vκ gene segments for complete cRS [4,6] and by showing that conserved Vκ cRS are cleaved.

To test the hypothesis that functional cRS are not conserved in Vκ gene segments, we conducted a global examination of mouse Vκ segments using the computational and experimental methods of our earlier study of V_H _cRS [25]. As in our study of V_H _cRS, we find that Vκ cRS are present and cleaved at multiple, conserved locations in Vκ gene segments. These cRS are conserved across Vκ gene families and are cleaved during the small pre-B cell stage of B-cell development. This study is the first to show that cRS are conserved within Vκ gene segments, and that these cRS are cleaved in vivo. Our findings support the hypothesis [25] that cRS are conserved in *Ig *V gene segments for a purpose(s) unassociated with the maintenance of self-tolerance.

## Results and Discussion

### Identification of cRS embedded in Vκ gene segments

To identify cRS in Vκ gene segments, we applied a statistical model of mouse RS to the 135 mouse Vκ gene segments and alleles listed in the Immunogenetics Information System (IMGT) reference directory set [4,6,31]. We previously used this analytic method to identify cRS in mouse V_H _gene segments and in a 212-kb control region of mouse chromosome 8 (accession AC084823) not subject to physiologic V(D)J recombination [6]. Our statistical model assigns a recombination information content (*RIC*) score to any RS-length DNA sequence beginning with the nucleotides CA; such sequences are referred to as potential cRS. DNA sequences of length 28-bp are assigned *RIC *scores based on the *RIC*_12 _model for RS with 12-bp spacers, while 39-bp sequences are assigned *RIC *scores based on the *RIC*_23 _model. Higher *RIC *scores indicate higher sequence similarities to mouse RS and are predictive of higher recombination efficiencies [1,4,6,25].

We have previously determined a threshold for 28-bp cRS of *RIC*_12 _≥ -45 using the *RIC *score of the functional cRS embedded in the 3H9 V_H _transgene [6,9,25]. 39-bp RS have a lower *RIC *score than 28-bp RS (*RIC*_23 _= -60 vs *RIC*_12 _= -40, respectively), thus we set a correspondingly lower threshold for the detection of 39-bp cRS of *RIC*_23 _≥ -65 [25].

We scanned for potential cRS on both DNA strands of each Vκ gene segment. Potential cRS found on the sense strand, and thus in the orientation of physiologic RS, are referred to as being in orientation 1 (O1). Potential cRS found on the antisense strand, and thus opposite in orientation to physiologic RS, are defined to be in orientation 2 (O2). Both strands of sequence AC084823 were also scanned. cRS in the strand listed in NCBI were arbitrarily assigned the O1 orientation, and cRS in the inverse complement sequence assigned to the O2 orientation.

The analyzed Vκ gene segments contained 6317 potential cRS with 12-bp spacers (12-cRS) (3729 in the O1 orientation and 2588 in O2) and 5995 potential 23-cRS (3628 in O1 and 2367 in O2) (Table 1). Of the potential 12-cRS identified, 75 (O1) and 81 (O2) had a *RIC*_12 _> -45, while 131 (O1) and 215 (O2) of the potential 23-cRS had a *RIC*_23 _>-65 (Table 1).

**Table 1:** The relative frequencies of 12- and 23-cRS in V_H_, Vκ, and control DNA.

	12-cRS				23-cRS			
	Orientation 1		Orientation 2		Orientation 1		Orientation 2	
	Potential 12-cRS	RIC_12_ ≥ -45	Potential 12-cRS	RIC_12_ ≥ -45	Potential 23-cRS	RIC_23_ ≥ -65	Potential 23-cRS	RIC_23_ ≥ -65
Vκ gene segments	3729	75 (.020)	2588	81 (.031)	3628	131 (.036)	2367	215 (.091)
V_H _gene segments	8647	223 (.026)	8976	299 (.033)	8312	135 (.016)	8109	302 (.037)
Ch. 8 (AC084823)	15401	259 (.017)	17480	321 (.018)	15401	701 (.046)	17478	831 (.048)

*RIC*_12 _and *RIC*_23_were computed for all 28-bp and 39-bp sequences beginning with a CA-dinucleotide in both O1 and O2 in V_H _and Vκ gene segments, and in a 212-kb portion of chromosome 8 (accession number AC084823). The number of sequences with *RIC *above -45 and above -65 are shown with the relative frequencies shown in parentheses.

### Vκ cRS are conserved in O2

We compared the relative frequencies of 12- and 23-cRS in Vκ gene segments with those present in control sequence AC84823 [25] and found that the relative frequencies of 12- and 23- Vκ cRS in the O2 orientation are significantly higher than in the AC84823 control (0.031 *vs*. 0.018; P = 10^-5 ^and 0.091 *vs*. 0.048; P = 10^-19^). In contrast, the frequencies of Vκ cRS in O1 do not differ from those in AC84823 (0.02 *vs*. 0.017; P = 0.17 and 0.036 *vs*. 0.046; P = 0.013, 12-cRS and 23-cRS, respectively) (Table 1). These biases for cRS in Vκ gene segments are unlike those of V_H _cRS, which contain significantly more O1 and O2 12-cRS and significantly fewer O1 and O2 23-cRS than AC84823 [25].

To examine further the differences between Vκ and V_H _cRS, we compared the distributions and orientations of Vκ cRS with those of the cRS present in V_H _gene segments [25]. Vκ gene segments exhibit significantly higher relative frequencies of 23-cRS in either O1 or O2 than do V_H _gene segments (0.036 *vs*. 0.016; P = 10^-11 ^and 0.091 *vs*. 0.037; P = 10^-26^, O1 and O2, respectively), whereas the relative frequencies of O1 and O2 12-cRS are not different between Vκ and V_H _gene segments (Table 1).

**Figure 1:**
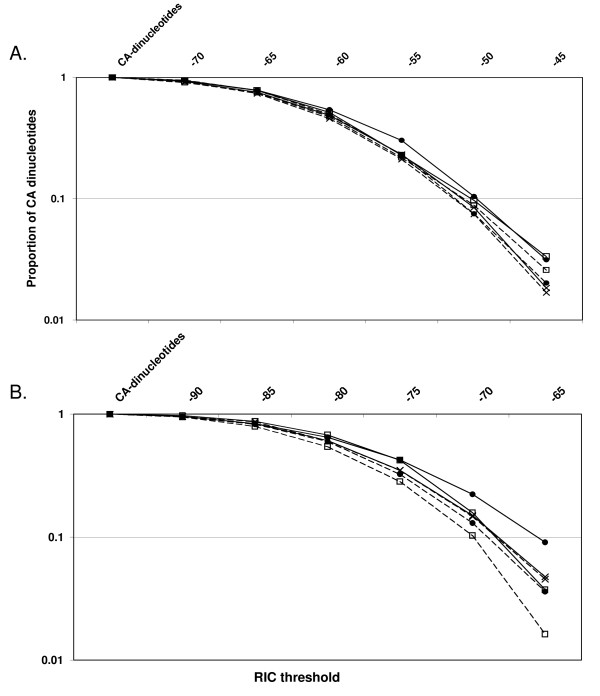
**The proportion of RS-length sequences with *RIC *scores above a given threshold**. For increasing *RIC *score thresholds, the number of RS-length sequences beginning with a CA-dinucleotide and with a *RIC *score above the threshold was divided by the total number of RS-length sequences beginning with a CA-dinucleotide. Thresholds are shown on the X-axis, and the corresponding proportions are shown on the Y-axis. The proportions of above-threshold sequences are plotted for both *RIC*_12 _(Figure 1A) and *RIC*_23 _(Figure 1B). Proportions from Vκ sequences are denoted by filled circles, those from V_H _sequences are denoted by an unfilled square, and those from chromosome 8 are denoted by an X. A dashed line indicates proportions of sequences in O1, while a solid line indicates those in O2.

Even though Vκ and V_H _gene segments and the AC084823 sequence exhibit similar relative frequencies of potential cRS, these frequencies diverge as *RIC *scores increase towards the threshold values associated with RS activity (Figure 1). O1 and O2 potential 12-cRS with *RIC*_12 _≥ -50 are more common in V_H _gene segments than in the AC084823 control (Figure 1A), while O1 and O2 potential 23-cRS with *RIC*_23 _> -70 are less common in V_H _gene segments than in the AC084823 control (Figure 1B) [25]. In contrast, of Vκ potential 12-cRS with *RIC*_12 _≥ -50, only those in O2 are more common than in AC084823 (Figure 1A), and O2 potential 23-cRS with *RIC*_23 _> -70 are more common in Vκ gene segments than in the control sequence (Figure 1B). As described above, at cRS *RIC *score thresholds, these differences are statistically significant.

Thus, while both V_H _and Vκ gene segments are significantly enriched for O2 12-cRS relative to the AC084823 control, V_H _gene segments appear to be selected for increased frequencies of 12-cRS and the suppression of 23-cRS, regardless of orientation, and Vκ gene segments appear to be under selection for O2 cRS, regardless of spacer length. These patterns of bias indicate that, relative to V_H _gene segments, Vκ segments are enriched for O2 23-cRS.

Given that the relative frequency of the 215 O2 23-cRS embedded within Vκ gene segments (0.091, Table 1) is much higher than the relative frequency of O2 23-cRS in V_H _gene segments or in AC84823, we examine the extent of Vκ O2 23-cRS conservation, explore whether their conservation can be explained by conservation of the encoded amino acid sequence, and determine whether they are cleaved.

### Vκ 23-cRS in O2 are conserved at multiple locations within Vκ genes and across Vκ gene families

**Figure 2:**
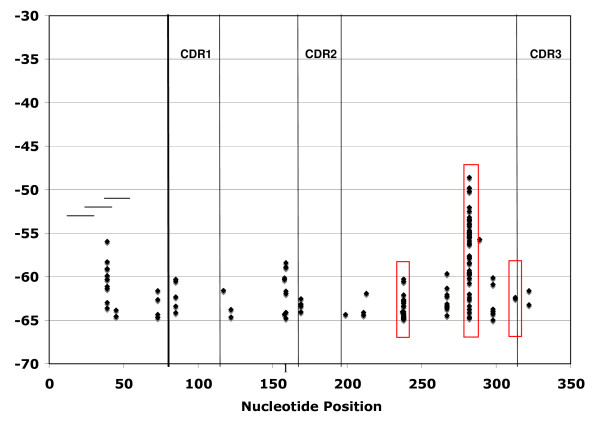
**O2 cRS are found at multiple locations within mouse Vκ gene segments**. RIC_23 _was computed for all 39-bp sequences beginning with a CA-dinucleotide in 135 mouse Vκ gene segments. *RIC*_23_scores are plotted for those 39-bp sequences in O2 and with *RIC*_23_>-65. *RIC*_23 _is shown on the Y-axis, and nucleotide position (IMGT numbering) within the gene segment is shown on the X-axis. Complementarity determining regions (CDR) are demarcated by vertical lines. The red rectangles indicate *RIC *scores located at the sites of cRS cleaved in vivo. The horizontal lines in FR1 indicate the location of the LM-PCR primers. Note that, because of the primer locations, cleavage events in FR1 are unlikely to be detected in our assay.

We first examined whether the locations of the 215 O2 23-cRS within Vκ gene segments were conserved across Vκ gene families. Indeed, a third (73/215; 33.95%) are located at nucleotide position 282 and one-fourth (53/215; 24.65%) at nucleotide position 238 in framework 3 (Figure 2). About 10% (22/215; 10.23%) of O2 23-cRS are located at nucleotide position 39 in framework 1, and the remaining 67 O2 23-cRS are distributed across 16 other locations (Figure 2). Importantly, only 3 cRS are located at nucleotide position 313, the position of the most highly conserved cRS in V_H _segments and of the cRS that mediates V_H _gene replacement [9,25].

Vκ 23-cRS in O2 are distributed across Vκ gene families. We found ≥ 1 cRS in 102 of the 135 Vκ gene segments analyzed, and most of these (66/102; 65%) contained multiple cRS. Further, cRS-containing Vκ gene segments were identified in 15 of the 19 Vκ gene families: 44 of the 215 O2 23-cRS are present in 27 of 31 Vκ4 gene segments; 34 are present in the 13 Vκ6 gene segments; 31 in the 10 Vκ3 gene segments; 25 in the 12 Vκ8 segments; 17 in the 5 Vκ2 gene segments; 17 in 5 of the 6 Vκ5 gene segments; 16 in 9 of the 11 Vκ12 gene segments; and, 13 are present in 11 of the 13 Vκ1 gene segments. The remaining 18 O2 23-cRS are found in families Vκ7 (4 cRS), Vκ10 (2 cRS), Vκ11 (2 cRS), Vκ13 (3 cRS), Vκ14 (3 cRS), Vκ15 (3 cRS), and Vκ16 (1 cRS).

### Vκ cRS are conserved independently of amino acid sequence

**Figure 3:**
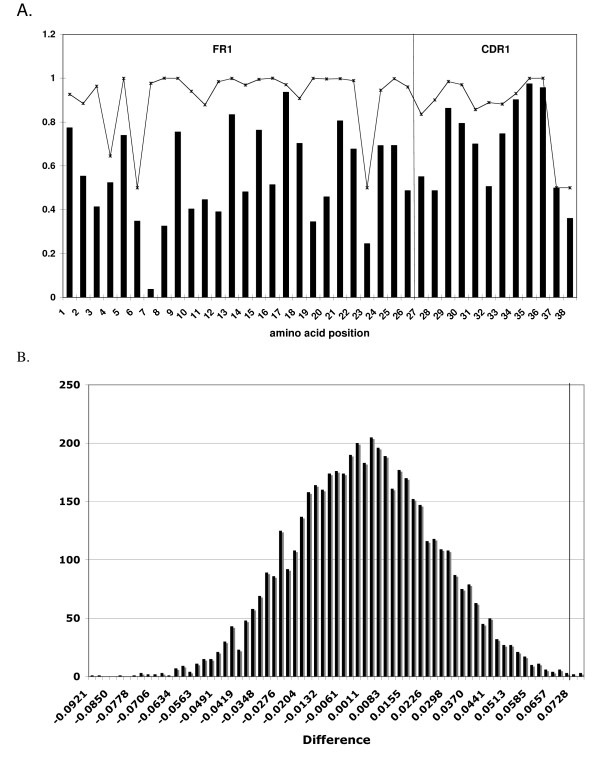
**Vκ cRS are conserved at the DNA level**. Using the Shannon entropy, the observed and maximum possible nucleotide diversity was calculated for each Vκ nucleotide position. Results for nucleotide positions occupying codon position 3 in FR1 and CDR1 are shown in panel A. Observed diversities are represented as bars, and maximum predicted diversities are represented by asterisks. The FR1-CDR1 boundary is marked by a vertical line. We computed the average difference between the observed and maximum possible DNA diversities for all nucleotide positions within codons that overlap one of the three conserved cRS () and for all nucleotide positions in FR codons not overlapping a cRS (). We simulated the null distribution for  - ; a histogram of 5000 simulated differences is shown in B. Difference is shown on the X-axis, and frequency is shown on the Y axis. The observed difference (0.073) is indicated by the vertical line.

The overrepresentation of O2 23-cRS at three conserved locations in Vκ gene segments and the wide distribution of these cRS across Vκ families, suggested that Vκ cRS are maintained by natural selection. Such selection might act directly on the cRS DNA sequences or indirectly, by selection for specific protein motifs encoded by cRS. To illuminate how cRS sequences might be conserved, we estimated the DNA sequence diversity in the 112 functional Vκ alleles contained in the IMGT reference directory (*H*_*O*_) and compared *H*_*O *_with the maximum diversity possible (*H*_*M*_) for a set of 112 nucleotide sequences with amino acid sequences identical to the observed (Figure 3).

*H*_*O *_was estimated by computing the Shannon entropy [32] at each nucleotide position employing the observed relative abundances of the four nucleotides. The maximum possible diversity *H*_*M *_was calculated by assuming, for each amino acid observed, a uniform distribution of codons for that amino acid among the subset of Vκ sequences with that amino acid at the relevant amino acid position. Thus, *H*_*M *_is calculated on the set of 112 simulated Vκ nucleotide sequences with amino acid sequences identical to the 112 observed Vκ sequences but with nucleotide sequences as diverse as possible, given the amino acid sequences. This calculation corresponds to the assumption that there is no constraint on evolution of Vκ DNA sequences beyond maintenance of the amino acid sequences. *H*_*O *_<*H*_*M *_suggests selection acting directly on DNA.

To determine whether the difference between *H*_*O *_and *H*_*M *_is greater for nucleotide positions within cRS than for other framework region (FR) nucleotide positions, we compared the average difference *H*_*M *_- *H*_*O *_for the 117 nucleotide positions in codons that overlap the three conserved cRS () with the average difference at all other FR nucleotide positions ().  was computed using nucleotide positions 1–39, 196–238, and 238–282, corresponding to amino acid residues 1–13, 66–80, and 80–94 (IMGT numbering). The average difference between *H*_*O *_and *H*_*M *_for the nucleotide positions within cRS ( = 0.198) was higher than that for the other FR nucleotide positions ( = 0.126). To determine whether the difference between the two averages ( -  = 0.073) was statistically significant, we randomly assigned each codon the label 'cRS' or 'FR', preserving the observed relative abundances of FR codons in and not in cRS, and computed  -  5000 times. Only 3 of 5000 permutations resulted in an  -  ≥ 0.073 (P = 0.0006) indicating that the observed difference  -  is statistically significant (Figure 3).

 is significantly greater than  because the difference between *H*_*O *_and *H*_*M *_is greater within cRS than for other FR nucleotide positions. Thus, the observed DNA sequence diversity (*H*_*O*_) for nucleotide positions within FR but not within cRS is relatively close to the maximum possible DNA sequence diversity (*H*_*M*_) that could be attained while conserving the amino acid sequences for the set of 112 functional Vκ gene segments. In contrast, the observed DNA sequence diversity for nucleotide positions within cRS is much less than the maximum possible diversity that could be attained while conserving the amino acid sequences, indicating selection on the DNA to a greater extent within cRS than within FR and beyond that required to maintain the necessary amino acid sequence. Thus, we conclude that the O2 23-cRS embedded within Vκ gene segments are not present as an artifact of amino acid conservation.

### Vκ cRS are cleaved *in vivo*

**Figure 4:**
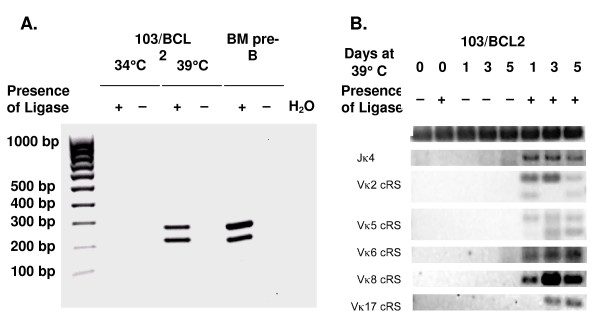
**Vκ cRS cleavage is detected in C57BL/6 bone marrow pre-B cells and 103/BCL2 cells**. LM-PCR was conducted with and without ligase on DNA extracted from pre-B cells of C57BL/6 mice or from 103/BCL2 cells after 0 and 3 days at 39°C (panel A) or from 103/BCL2 cells after 0, 1, 3, and/or 5 days at 39°C (panel B). Vκ 6 cRS SE (expected product size: 217 bp and 277 bp) were detected in 103/BCL2 cells and C57BL/6 bone marrow pre-B cells (panel A). LM-PCR product from primary Jκ rearrangements demonstrates that 103/BCL2 cells are rearranging their κ loci (panel B). Vκ cRS SEs were detected in Vκ2, Vκ5, Vκ6, Vκ11, and Vκ17 gene segments in 103/BCL2 cells (panel B). CD14 PCR demonstrates the equivalence of genomic template.

To determine if the Vκ O2 23-cRS identified by *RIC *scores are cleaved *in vivo*, we performed ligation-mediated PCR (LM-PCR) [33] to amplify Vκ cRS signal ends (SE) recovered from 103/BCL2 cells and small pre-B cells from the bone marrow of C57BL/6 mice (Figure 4). LM-PCR is a standard assay used to demonstrate RAG-mediated cleavage at RS and cRS heptamers [33]. RAG expression in 103/BCL2 cells is temperature dependent. At 34°C, 103/BCL2 cells proliferate, RAG1 and RAG2 proteins are minimally expressed, and *Igκ *rearrangements are undetectable [34]. At 39°C, 103/BCL2 cells enter growth arrest, RAG1 and RAG2 expression is upregulated, and *Igκ *rearrangements are induced [34]. To control for potential LM-PCR artifacts, we used genomic DNA from 103/BCL2 cells cultured at 34°C and 39°C as LM-PCR templates, in addition to DNA from sorted pre-B cells (Figure 4). To determine the extent of functional O2 cRS in gene segments from the Vκ2, Vκ5, Vκ6, Vκ8, and Vκ17 families, we designed a series of Vκ family-specific PCR primers and used a standard intronic LM-PCR [25] to detect primary Jκ SE as a positive control. The Vκ primers are designed such that only O2 cRS are detected.

LM-PCR amplicands representing RAG- and ligase-dependent Vκ cRS SE cleavage products were readily detected in both 103/BCL2 and small pre-B cells (Figure 4A). The dual products recovered from both 103/BCL2 and bone marrow cells using Vκ6 and Vκ8 family primers represent cleavage at nucleotide positions 282 (220-bp fragment) and 342 (280-bp fragment) in Vκ6 gene segments (Figure 4A) and at positions 288 and 327 in Vκ8 gene segments (data not shown). Similarly, LM-PCR amplifications of genomic DNA from 103/BCL2 cells using five sets of Vκ family-specific primers indicated that ≥ 1 cRS is present and cleaved in V gene segments belonging to the Vκ2, -5, -6, -8, and -17 gene families (Figure 4B), all of the families for which cleavage was assayed.

To ensure that these LM-PCR amplification products represented *bona fide *Vκ cRS SE, the LM-PCR amplicands were gel-purified, cloned, and sequenced (Tables 2 and 3). Of 101 sequences obtained, 82 represent Vκ gene segments ending precisely at blunt, double-strand ends (Tables 2 and 3). Almost one quarter of the 82 Vκ LM-PCR products (19/82, 23%) have unique nucleotide sequences or were obtained from independent PCR reactions and were therefore determined to be the product of independent cleavage events. The 39 nucleotides immediately 5' of the cleaved ends are shown for these 19 LM-PCR products in Table 2. The two sequences from Vκ8 gene segments indicate cleavage at non-CA nucleotides (Table 2). The remaining 17 sequences represent unique cleavage events precisely at Vκ cRS heptamers (Table 2). The 17 unique cRS cleavage events occurred at 5 different Vκ nucleotide positions (232, 238, 282, 313, and 342) from 6 distinct Vκ gene families (Vκ2, Vκ5, Vκ6, Vκ11, Vκ14, and Vκ17; Table 2). Although no primer set was used to amplify gene segments of the Vκ11 and Vκ14 families, the Vκ8 primer set matches to both Vκ11 (17 of 18 nucleotides) and Vκ14 (15 of 18 nucleotides) genes and resulted in the amplification of a single Vκ11 cRS SE product and two Vκ14 cRS SE products (Table 2).

**Table 2:** Vκ-embedded cRS cleaved in 103/BCL2 cells and pre-B cells from RAG2:GFP and C57BL/6 mice.

Total Number of Sequences	101
Number of Sequences from Vκ Gene Segments	82
Number of Sequences from Independent Cleavage Events	19
Number of Sequences from Cleavage at VκcRS	17
Number of Sequences from Cleavage at Vκ-embedded cRS	10

LM-PCR products from 103/BCL2 cells and from pre-B cells isolated from RAG2:GFP knock-in mice and C57BL/6 mice were sequenced and aligned to sequences from the IMGT reference directory set to identify products from cleavage at Vκ-embedded cRS.

**Table 3:** Vκ cRS cleaved in 103/BCL2 cells and pre-B cells from RAG2:GFP and C57BL/6 mice.

Cell Type	Position	N	cRS sequence	Vκ gene	*RIC*_23_
103BCL2	232	1	cactgaa *cctgtttgggactcctgaggcca *gattggaca	**2–116**	-76.53
103BCL2	238	1	cactAct *ggagaaCcgggatgggactccag *gacgaagag*	17–127	-72.20
103BCL2	282	2	cacagtg *ctgatggtgaaagtgaaatccgt *cccatatcc	6–32	-52.01
103BCL2	282	1	cacactg *ttgatactgagagtgaaatctga *ccctgatcc	5–39	-54.66
103BCL2	282	1	cacactg *ttgatactgagagtgaaatctgt *ccctgatcc	5–45	-54.66
BM pre-B	282	2	cacagtg *ctgatggtgaaagtgaaatccgt *cccatatcc	6–32	-52.01
BM pre-B	288	1	agcatgc acattgctaatggtgagagtgaa gtctgtccc	8–26	NA
103BCL2	313	1	cacagta *ataaacacccacatcctcagcct *ccactctgc	**2–116**	-62.49
103BCL2	313	1	cacagta *ataaacacccacatcctcagcct *ccactctgc	2–109	-62.49
103BCL2	327	1	gccatga ttgtgctgacagtaataaactgc taggtcttc	8–18	NA
103BCL2	342	1	cactgtg *tgaggccagctgttactctgttg *acagaaata	5–45	-65.18
103BCL2	342	1	cactgtg *ggagggagatacctatgctgtag *acagaaata	**11–106**	-69.65
BM pre-B	342	2	cactgtg *ggaggagagctataatcctgctg *acagaaata	6–32	-66.82
103BCL2	342	1	cactgtg *ggaggagagctataatcctgctg *acagaaata	6–32	-66.82
103BCL2	342	1	cactgtg *ggaggaaactcataaaactgtag *acagtaata	14–130	-70.3
BM pre-B	342	1	cactgtg *ggaggaaactcataaaactgtag *acagtaata	14–130	-70.3

LM-PCR products from 103/BCL2 cells and from pre-B cells isolated from RAG2:GFP knock-in mice or from C57BL/6 mice were sequenced and aligned to sequences from the IMGT reference directory set to identify the germline gene segment. Where matches to IMGT sequences were not found, the LM-PCR products were aligned to germline Vκ gene segments in NCBI (indicated in bold). The source, location, number of observations, cRS sequence, Vκ gene segment, and cRS sequence RIC score are shown for each independent cleavage event. cRS sequences are written in heptamer-to-nonamer orientation, and nucleotide positions using IMGT numbering indicate the location of the first heptamer nucleotide.

* The LM-PCR product shows 2 mismatches to the genomic cRS sequence (*cactGctggagaaTcgggatgggactccaggacgaagag*), indicated with capital letters. We attribute this difference to sequencing error.

Three of the 5 positions at which Vκ cRS cleavage was detected (nucleotide positions 238, 282, and 313) are cRS predicted by *RIC*, and two of these (positions 238 and 282) are the most frequent sites for Vκ O2 23-cRS (Figure 2). Position 342 is downstream of the coding region of Vκ gene segments (IMGT database) and was not considered in our computational analysis. Interestingly, cRS SE at position 342 were observed in pre-B and 103/BCL-2 cells (Figure 4, Table 2). Cleavage occurred at the 3' end of the physiologic RS heptamer such that the last 3 nucleotides (GTG) of the physiologic heptamer are the first 3 nucleotides (CAC) of the cRS heptamer. Seven of 17 unique Vκ cRS cleavage events were at position 342; the remaining 10 were at cRS embedded within the Vκ gene segment. Thus, 17 of the 19 cleavage events we observe (89%) represent cleavage at cRS, and 59% of these represent cleavage at Vκ-embedded cRS (Tables 2 and 3).

Six of the 10 Vκ-embedded cRS cleavage events were at nucleotide position 282 (Table 2), the most conserved location for O2 23-cRS identified by *RIC*. Cleavage at this cRS was identified in the Vκ6–32, Vκ5–39, and Vκ5–45 gene segments. The remaining 4 Vκ-embedded cRS cleavage events were distributed as follows: 1 at Vκ nucleotide position 232 in Vκ2–116, 1 at position 238 in Vκ17–127, and 2 at position 313 in the Vκ2–109 and Vκ2–116 gene segments (Table 2). Thus, we observe cleavage events occurring both at the same location across different Vκ gene families, and at different locations within the same gene family.

Vκ6 cRS SE and Jκ4 SE are approximately equally abundant in recombinationally active 103/BCL2 cells (Figure 4 and data not shown). Given that the Vκ6 family comprises eight or nine gene segments (IMGT database) and that each of these likely contain at least two functional cRS (Table 2), we estimate the rate of Vκ6 cRS cleavage to be 5% – 13% of Jκ4 RS.

### Vκ cRS SE are detected only in pre-B cells

To identify the developmental stages in which Vκ cRS are cleaved, we isolated genomic DNA from highly enriched (>95%) populations of pro-B, pre-B, and immature B cells sorted from the bone marrow of C57BL/6 mice and congenic RAG2:GFP animals [25]. We previously demonstrated that V_H _cRS SE are present in pro-B cells but not in pre-B or immature B cells from the bone marrow of RAG2:GFP mice [25]. In this study, J_H _RS SE were detected only in pro-B cells, Jκ RS SE only in pre-B cells, and TCR Dβ RS SE were not detected in any B-cell population [25].

**Figure 5:**
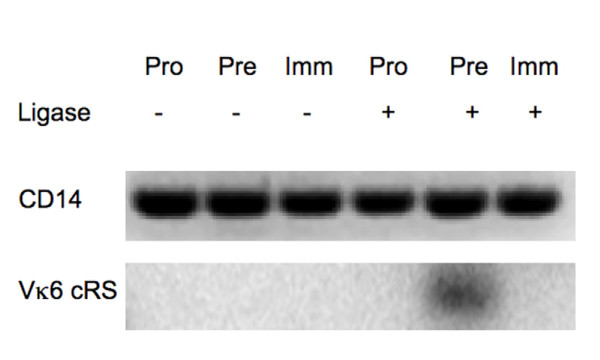
**Vκ cRS cleavage is detected only in pre–B cells from RAG2:GFP knock-in mice**. LM-PCR was conducted on pro-, pre-, and immature B cells from RAG2:GFP knock-in mice [25]. These sorted cells were shown in [25] to have the appropriate lineage and developmental restrictions of Tdt and RAG1 expression, and of J_H _and Jκ SE. Samples from these same sorted cells were used to amplify SE in Vκ 6 gene segments. Cleavage at Vκ 6-embedded cRS was detected only in pre-B cells. CD14 amplification demonstrates the equivalence of genomic template.

To determine if Vκ cRS are cleaved in vivo and to identify the developmental stage in which cleavage occurs, we isolated genomic DNA from the samples of bone marrow pro-, pre-, and immature B cells sorted in the previous study [25]. The genomic DNA was used as template for LM-PCR to detect cleavage of O2 Vκ cRS in the Vκ6–32 gene. We targeted this Vκ gene segment because it contains multiple cRS (Figure 4) with the highest *RIC *scores of the cRS for which SE were detected in 103/BCL2 cells (Table 3). Ligase-dependent, Vκ6–32 cRS SE could be detected at nucleotide positions 282 and 342 (Figure 4 and Tables 1 and 2) in pre-B, but not pro-B or immature B cells (Figure 5). These LM-PCR products were validated as Vκ6–32 cRS SE by sequencing (Table 3). Thus, Vκ cRS appear to be cleaved *in vivo *during the developmental stage that is permissive for primary *Igκ *Vκ → Jκ rearrangements.

## Conclusion

The adaptive immune system has evolved to generate a diverse antigen-receptor repertoire. One mechanism of somatic diversification is V(D)J recombination, a process that joins antigen-receptor V, D, and J gene segments by initiating double-strand breaks at RS flanking the gene segments (for a review, see [1]). RS at locations other than the boundaries of V, D, and J segments have been identified at both the *Igh *and *Igκ *loci [22,23]. Until recently, cRS in the *Igh *locus were thought to be limited to the 3' end of V_H _gene segments where cRS can mediate V_H _gene replacement [7,9,22-24]. V_H _gene replacement can participate in a form of receptor editing at the heavy chain locus, which otherwise is incapable of secondary rearrangements that follow the 12/23 rule [7]. It has been proposed that the utility of receptor editing is sufficient to drive the evolutionary conservation of V_H _cRS [7]. There is mounting evidence, however, that at least some receptor editing is antigen-independent, and that the conservation of Ig V_H _cRS may result from other selective pressures.

The earliest evidence that the regulation of V_H _replacement is independent of BCR-specificity came from studies [35-37] that demonstrated frequent V_H _replacement in mice transgenic for non-autoreactive heavy chains. These data suggested that selection for V_H _cRS includes the capacity for increasing BCR diversification, in addition to self-tolerance [8,35]. We subsequently showed that V_H _cRS SE were detected only in pro-B cells, including the pro-B cells of μMT mice which can not assemble functional BCR [25,38]. Together, these results support the notion that V_H _gene replacement may not be driven by the recognition of antigen.

Koralov et al. [39] demonstrated that, in transgenic mice homozygous for nonproductive heavy-chain rearrangements, V_H _replacement events are only three times more frequent than direct V_H _to J_H _joining, in violation of the 12/23 rule. These results demonstrate the inefficiency of cRS-mediated V_H _replacement and beg the question: *How can such an inefficient mechanism for rescuing autoreactive B cells increase fitness sufficiently to maintain V*_H_*cRS conservation? *If V_H _cRS are conserved to mediate V_H _replacement, shouldn't V_H _replacement at cRS be much more efficient than rearrangements in violation of the 12/23 rule? The results of Koralov et al. [39] suggest that while V_H _replacement may be mediated by V_H _cRS, their conservation is unlikely to result only from their role in V_H _replacement.

Unlike the cRS associated with *Igh*, the cRS previously identified in *Igκ *loci were not embedded in Vκ gene segments but sited in the Jκ-Cκ intron and 3' of Cκ and mediated locus inactivation [11,17,20-22]. The cRS located in the Jκ-Cκ intron are known as IRS (IRS1 and IRS2), while the cRS found 3' of Cκ is named the kappa deleting element (kde) in humans and RS in mice. For clarity, we reserve 'RS' for signals adjacent to V, D, and J gene segments, and refer to the signal 3' of Cκ in mice as RS_κ3_.

The structure of the *Igκ *locus allows for secondary Vκ → Jκ rearrangements. Thus, if antigen-driven receptor editing is the primary force behind conservation of V-gene cRS [7,9], Vκ gene segments should not be selected for embedded cRS. Fanning et al. [8] noted the presence of a partial heptamer motif (CACA) in Vκ gene segments at a location orthologous to the 3' V_H _cRS, but to date, there has been no systematic attempt to identify potential cRS at other sites within Vκ gene segments or to determine their function. The determination of cleaved cRS within Vκ gene segments is an important first step in identifying their physiologic role(s) and resolving the selective forces that maintain their conservation.

To determine whether the *Igκ *locus contains active cRS embedded in functional Vκ gene segments, we conducted a computational scan for cRS in Vκ gene segments and evaluated their functionality using LM-PCR. Our results indicate that, despite the capacity for repeated secondary *Igκ *rearrangements, functional Vκ cRS have been evolutionarily conserved. Vκ cRS are primarily conserved in an orientation (O2) opposite to physiologic Vκ 12-RS and have 23-bp spacers (Table 1 and Figure 1). This conserved orientation and spacer size mirrors our earlier demonstration that conserved V_H _cRS are oriented opposite to physiologic V_H _23-RS and contain 12-bp spacers [25].

As with V_H _cRS, Vκ cRS are conserved at multiple sites in Vκ gene segments and across Vκ gene families. Although our genomic scan identified relatively few Vκ cRS at positions analogous to the 3' V_H _cRS (nucleotide position 313, IMGT numbering) that mediate V_H _replacement (Figure 1), we did observe two cRS SE at this location, both in Vκ2 gene segments (Table 2). Of the 10 unique cleavage events at Vκ-embedded cRS, 8 represent cRS SE ≥ 30 nucleotides upstream of complementarity determining region (CDR) 3 (Table 2). V gene replacement (Vκ → VκJκ) at one of these embedded cRS would result in substantially lengthened variable-region product that would be unlikely to produce a typically folded L-chain protein. The conservation of functional cRS at such sites in Vκ gene segments in a locus capable of secondary Vκ → Jκ rearrangements implies a function distinct from immunological tolerance.

cRS previously identified at the *Igκ *locus (IRS1, IRS2 and kde/RS_κ3_) mediate rearrangement events that inactivate the locus and may serve to ensure *Igκ *allelic exclusion or activation of the *Igλ *loci (reviewed in [40]). Rearrangements between kde/RS_κ3 _and IRS result in the deletion of Cκ and rearrangements between kde/RS_κ3 _and Vκ RS result in the deletion of Jκ and Cκ [21,41]. It is possible that the O2 Vκ 23-cRS likewise participate in these inactivation rearrangements, as recombination between IRS and O2 Vκ 23-cRS would result in deletion or inversion of the Jκ gene segment cluster.

Inactivating rearrangements involving IRS and kde/RS_κ3 _have been implicated in antigen-induced receptor editing (reviewed in [22]), and Kiefer et al. [42] observed RS_κ3 _cleavage in IgM^- ^BM pre-B cells, IgM^low ^immature BM B cells, and in IgM^low^IgD^+ ^splenic T3/T3' B cells. Our results indicate that cleavage of O2 Vκ 23-cRS is confined to the IgM^-^, small pre-B compartment (Figures 4 and 5). We conclude that either Vκ cRS SE are rare relative to RS_κ3 _SE, or that Vκ cRS SE are not present in immature B cells (perhaps because the cRS themselves are not accessible) and, consequently, may be unrelated to antigen-driven receptor editing. In either case, despite their frequency and function, Vκ cRS appear to play a less significant role in antigen-driven genomic change than do IRS and kde/RS_κ3_.

The similarities between the V_H _and Vκ cRS suggest that these DNA motifs are conserved for a common function. Both cRS types are conserved at multiple locations, and both are conserved with an orientation and spacer length opposite to the corresponding physiologic V-associated RS. Both sets of cRS are cleaved coincidentally with the physiologic RS in the same locus. That is, V_H _cRS are cleaved in pro-B cells and Vκ cRS are cleaved in pre-B cells. We consider below possible mechanisms for conservation of these V-gene cRS in the *Igh *and *Igκ *loci.

First, V_H _and Vκ cRS could be conserved to inactivate the *Igh *and *Igκ *loci. If so, this inactivation might help to ensure allelic exclusion, as evidence indicates that V_H _[25] and Vκ cRS SE (Figure 5) do not depend on the generation of a functional B-cell receptor. Inactivation of the *Igκ *locus would increase the proportion of λ-expressing B cells and could act to increase the diversity of the BCR repertoire. A similar argument cannot be made for the *Igh *locus as there is no alternative locus. Furthermore, the frequency of IRS-to-kde/RS_κ3 _rearrangements mitigates any need for V-embedded cRS for inactivation at the κ locus. Thus, we doubt that the selection pressure resulting from locus inactivation via V cRS cleavage is sufficient to result in conservation of the cRS.

We previously suggested that V-embedded cRS could function to form hybrid V gene segments thereby creating combinatorial diversity beyond that created through the combination of V, D, and J or V and J gene segments [25]. While the results are controversial, there is evidence for such hybrid heavy chain V genes [43,44]. Given that both V_H _and Vκ cRS are conserved in opposite orientation and with the complementary spacer length to physiologic, V-associated RS, we propose that V-embedded cRS may be conserved to recombine with physiologic RS to form hybrid V genes. Under this model, hybrid V gene formation would proceed by a two-step process. Recombination of an O2 Vκ 23-cRS to the same Vκ gene segment's physiologic RS would result in deletion of the intervening nucleotides and generation of a SJ intermediate. A second recombination event could then occur between the RS of the SJ and an O2 Vκ 23-cRS located at the same or a nearby nucleotide position in a downstream Vκ gene segment. This two-step rearrangement would be rare, but would result in a novel, hybrid Vκ gene segment of approximately normal length. In particular, utilization of O2 23-cRS located in FR2 would create CDR1 – CDR2 combinations not present in the germline.

An alternative hypothesis to the conservation of cRS for their recombinogenic potential is that the nucleotide sequences are conserved to maintain appropriate V region amino acid sequences, and the corresponding recombinogenic potential is a coincidence. We present evidence that the conservation of O2 cRS embedded in V_H _and Vκ is not explained by the need to maintain V region amino acid sequences ([25] and Figure 3). In V_H _gene segments, the second, third, and fourth nucleotides of the 3' cRS (...TGTG) encode the conserved Cysteine at amino acid position 104 (Cys_104_), while the codon for the conserved Cysteine at amino acid position 23 (Cys_23_) is not part of any known cRS. Cysteine is degenerately encoded, and we find that only 38% of Cys_23 _are encoded by TGT [25]. Ninety-eight percent of Cys_104 _are encoded by TGT, however, providing evidence for selection pressure to maintain the recombinogenic potential of the 3' V_H _cRS [25]. Similarly, analysis of FR codons in Vκ gene segments shows that codon diversity at cRS is reduced relative to the maximum possible to a significantly greater extent than at any other FR site (Figure 3), a finding that implies stringent selection against synonymous nucleotide substitutions in the cRS. The absence of synonymous mutations is important given that the predicted recombinogenic potential of most conserved (116/128) O2 Vκ 23-cRS could be eliminated by a single, synonymous nucleotide substitution (data not shown). Of the remaining 12 cRS, the recombinogenic potential for 10 of them would be significantly reduced (>90%) by one synonymous nucleotide substitution (data not shown). Thus, while nucleotide substitutions in cRS motifs that eliminate efficient recombination without altering Vκ amino acid sequence are potentially frequent, they are rare or absent in the genome. We conclude that there is evolutionary selection for V_H_- and Vκ-embedded O2 cRS.

Another alternative hypothesis to the conservation of V-gene cRS for their recombinogenic potential is that the cRS nonamers are conserved for nucleosome positioning. Consensus RS nonamers may contribute to nucleosome positioning and influence RS accessibility to the V(D)J recombinase [45]. While the cRS nonamers may influence nucleosome positioning, this property is unlikely to explain conservation of V-gene cRS. First, *RIC *scores are based on the complete cRS sequence, and above-threshold *RIC *scores would not result from conserved nonamer motifs alone. Second, cleaved Vκ cRS (Table 2) do not contain consensus nonamers and lack the stretch of adenosine nucleotides thought to be responsible for nucleosome positioning [45,46]. Thus, it is unlikely that selection for nucleosome positioning motifs has resulted in the maintenance of functional Vκ cRS.

We provide the first exhaustive search using a rigorous method for cRS embedded in Vκ gene segments. We demonstrate not only that Vκ cRS are conserved, but also that they are cleaved in vivo. We show that the patterns of conservation for Vκ cRS are analogous to those for V_H _[25], namely that the V-embedded cRS are conserved with an orientation and spacer length opposite to that for V-associated RS in the same locus. We provide evidence that these V-embedded cRS are not conserved as a consequence of selection pressure to maintain V region amino acid sequence and explore several possible explanations for their conservation. While the role of these V-gene cRS is not yet clear, their conservation in both V_H _[25] and Vκ gene segments implies a substantial evolutionary benefit to their presence.

## Methods

### Identification of Vκ cRS

To identify cRS in Vκ gene segments, we computed the RS information content (RIC) score for 28- and 39-bp segments in the 135 mouse Vκ gene segments available in the Immunogenetics Information System (IMGT) reference directory set [47]. RIC is based on the position-specific nucleotide combinations present in a sequence and the relative frequency of these nucleotide combinations in the set of mouse physiologic RS; sequences with nucleotide combinations frequent in mouse physiologic RS have a high *RIC *score [4]. We have previously demonstrated that *RIC *scores can be used to identify RS and cRS and are predictive of recombination efficiency [4,6].

We used *RIC *scores to determine the location and number of 12- and 23-cRS in mouse Vκ gene segments in both orientations and compared the corresponding relative frequencies with those previously reported for cRS in mouse V_H _gene segments and in a 212-kb region of mouse chromosome 8 (NCBI accession AC084823). Statistical significance was determined using Chi-square tests.

### Estimation of nucleotide diversity

To estimate the nucleotide diversity at each Vκ framework region position, we computed the Shannon entropy [32] at position *i * where *p*_*i*, *j *_is the probability of nucleotide *j *at position *i *and *C *is any constant. We estimated *p*_*i*, *j *_as *n*_*i*, *j*_/*N*_*i *_where *n*_*i*, *j *_is the number of nucleotides of type *j *observed at position *i *and *N*_*i *_is the total number of functional Vκ sequences with a known nucleotide at position *i*. The IMGT reference directory set used for this analysis contained a total of 112 functional Vκ sequences.

To estimate the maximum possible diversity at nucleotide position *i*, *p*_*i*, *j *_was estimated by  where *N*_*i*, *k *_is the number of functional Vκ sequences with amino acid *k *encoded by the codon of which nucleotide position *i *is part, *m*_*k *_is the number of possible codons for amino acid *k*, and *m*_*i*, *j*, *k *_is the number of *k *codons with nucleotide *j *at codon position *i*. For example, when nucleotide position *i *is the second position of a codon encoding either Leucine or Phenylalanine, *m*_*L *_= *6*, *m*_*F *_= *2*, *m*_*i*, *T*, *L *_= *2*, *m*_*i*, *C*, *L *_= *4*, and *m*_*i*, *T*, *F *_= *2*. This equation assumes a uniform distribution of codons for each amino acid and preserves the observed relative abundances of amino acids.

For each nucleotide position, we computed the difference between the maximum possible entropy and the observed entropy. We computed the average difference over all nucleotide positions within codons for which at least one codon position is within one of the three conserved cRS () and compared this difference with the average over all other framework region nucleotide positions (). We then computed the difference  - .

To simulate the distribution of  -  under the null hypothesis that, for any nucleotide position, being located within one of the conserved cRS does not affect the difference between the observed and the maximum possible entropy, we randomly assigned each codon to either a cRS or non-cRS position according to the observed relative frequencies of codons within cRS and not, and computed  -  for each randomization. We performed 5000 randomizations.

### 103/BCL2 cells

103/BCL2 cells were cultured at 34°C and 39°C as previously described [6]. At 39°C, 103/BCL2 cells initiate apoptosis, and their viability ranges from 50%–100% after 1 to 5 days at 39°C [34]. To isolate genomic DNA from viable cells only, we enriched viable 103/BCL2 cells by density-gradient centrifugation with Lympholite-M (Accurate Chem).

### Mouse bone marrow B cells

C57BL/6 mice were purchased from Jackson laboratory. RAG2:GFP mice [48] were obtained from F.W. Alt (Harvard University, Boston, MA). All mice were housed in specific pathogen-free conditions at the Duke University Medical Center Vivarium, and all experiments using animals were reviewed and approved by the Institutional Animal Use and Care Committee of Duke University. Pro- (B220^lo^CD43^+^IgM^-^IgD^-^Lin^-^7AAD^-^), pre- (B220^lo^CD43^-^IgM^-^IgD^-^Lin^-^7AAD^-^), and immature (B220^lo^CD43^-^IgM^+^IgD^-^Lin^-^7AAD^-^) B cells were sorted from RAG2:GFP as previously described [25]. Lin refers to the lineage markers Mac-1, Gr-1, TER-119, CD4, and CD8 [25].

### LM-PCR

Genomic DNA was extracted from 103/BCL2, C57BL/6, and RAG2:GFP bone marrow B cells and ligated to the BW-LC linker [6,33]. Ligated genomic DNA from 2 or 4 × 10^3 ^bone marrow B cells or 4 × 10^4 ^103/BCL2 cells was used in each PCR. B cell lineage and developmental stage were demonstrated by amplification of J_H_, Jκ and Dβ RS signal ends [25]. Vκ cRS SE were amplified by a nested LM-PCR: the primary LM-PCR included High Fidelity Platinum Taq (Invitrogen), Advantage 2 (BD Clontech), the BW-LCH primer [25], and a degenerate Vκ primer (VκcRS below). The amplification program included melting at 94°C and extension at 68°C. Annealing was performed at 60°C for 5 cycles, 58°C for 4 cycles, 56°C for 3 cycles, and 54°C for 18 cycles. Denaturation, annealing, and extension occurred for 30 seconds each, with the exception of the initial denaturation (2 minutes) and final extension (10 minutes). In *lieu *of MgCl_2_, as directed by the manufacturer, 2 mM MgS0_4 _was used with High-Fidelity Platinum Taq.

The nested LM-PCR was performed with 10% of the primary LM-PCR product as template, High Fidelity Platinum Taq or Advantage 2, BW-LCH, and Vκ family specific primers. Nested LM-PCR conditions were the same as for the primary LM-PCR, except that the amplification program was modified with annealing only at 54°C for 27 cycles or at 56°C for 25 cycles. LM-PCR products from 103/BCL2 cells were electrophoresed over 1% agarose gels and stained with SYBR-Green (Molecular Probes) at a 1:10^4 ^dilution. LM-PCR products from RAG2-GFP sorted B cells were electrophoresed over 1% agarose gels, stained with SYBR-Green, and then transferred to nylon membranes [25]. The nylon membranes were then hybridized with a Vκ degenerate probe, radio-labeled with P^32^, to identify double-strand breaks in Vκ gene segments [25]. In some experiments, LM-PCR products for 103/BCL2 and C57BL/6 pre-B cells were electrophoresed over 1.5% agarose gels and stained with ethidium bromide. LM-PCR products were gel-purified and cloned into the pCR2.1 vector as described [6]. Clones were sequenced at the Duke University DNA Analysis Facility.

Oligomer sequences for the primers are VκcRS (5'-ATTGTGATG ACCCAGACTCC-3'), Vκ2 (5'-CAGTCACTCTTGGAACATCA-3'), Vκ5 (5'-GACTCAGTCTCCAGCCAC-3'), Vκ6 (5'-TTGTATCAGCAGGAGACAGG-3'), Vκ8 (5'-GACACAGTCTCCAT CCTC-3'), and Vκ17 (5'-CAGCATCCCTGTCCATGGCTA-3'). The Vκ degenerate probe is (5'-GSTTCAGTGGCAGTGGRTCTGGRAC-3').

### PCR

CD14 amplification was performed as in [25].

## Abbreviations

cRS: cryptic recombination signal; V: variable gene segment; D: diversity gene segment: J: joining gene segment; RS: recombination signal; bp: base pairs; RAG: recombinase activating gene; BCR: B cell receptor; LM-PCR: ligation-mediated PCR; *RIC*: recombination information content; O1: the orientation of physiologic RS relative to their associated gene segment; O2: the opposite orientation from O1; 12-cRS: cRS with 12-bp spacers; 23-cRS: cRS with 23-bp spacers; *H*_*O*_: Shannon entropy computed from observed relative nucleotide frequencies; *H*_*M*_: Shannon entropy computed from relative nucleotide frequencies that maximize the nucleotide diversity while preserving the relative amino acid frequencies; FR: framework region; : the difference between *H*_*O *_and *H*_*M *_averaged over nucleotide positions within codons that overlap with cRS nucleotide positions; : the difference between *H*_*O *_and *H*_*M *_averaged over nucleotide positions within FR codons that do not overlap with cRS; SE: signal ends; C: constant region exon; IRS1: κ-locus intronic recombination signal 1; IRS2: κ-locus intronic recombination signal 2; kde: kappa deleting element; RS_κ3_: cRS 3' of Cκ in mice; CDR: complementarity determining region; Cys_104_: cysteine at amino acid position 104; Cys_23_: cysteine at amino acid position 23.

## Authors' contributions

AEL and MD carried out the experiments on 103/BCL2 cells and RAG2:GFP mice. MK carried out the experiments on 103/BCL2 cells and C57BL/6 mice. AEL and LGC carried out the computational analyses and drafted the manuscript. GK provided significant revisions to the manuscript. GK and LGC conceived of the study and participated in its design and coordination. All authors read and approved the final manuscript.
